# Axial length acquisition success rates and agreement of four optical biometers and one ultrasound biometer in eyes with dense cataracts

**DOI:** 10.1186/s40662-023-00352-3

**Published:** 2023-09-01

**Authors:** Pedro Tañá-Rivero, Salvador Aguilar-Córcoles, Pedro Tañá-Sanz, Santiago Tañá-Sanz, Robert Montés-Micó

**Affiliations:** 1 Oftalvist, C/Angel Lozano 11, 03001 Alicante, Spain; 2 Optics and Optometry and Vision Sciences Department, University of Valencia, Valencia, Spain

**Keywords:** Dense cataracts, Optical biometry, Swept-source optical coherence tomography, Partial coherence interferometry, Ultrasound biometry

## Abstract

**Background:**

To evaluate the axial length acquisition success rates and agreement between various biometric parameters obtained with different biometers in dense cataracts.

**Methods:**

Fifty-one eyes were measured using Anterion®, Argos® and IOLMaster® 700 swept-source optical coherence tomography (SS-OCT) biometers, a Pentacam® AXL partial coherence interferometry (PCI) biometer, and an OcuScan® RxP ultrasound biometer. We measured keratometry (K1, flattest keratometry and K2, steepest keratometry), white-to-white (WTW), anterior chamber depth (ACD), lens thickness (LT) and axial length. Cataracts were classified according to the Lens Opacities Classification System III grading system, the dysfunctional lens index (DLI) and Pentacam® nucleus staging (PNS) metrics. Percentage of acquisition success rate and a Bland–Altman analysis for the agreement between biometers were calculated.

**Results:**

The mean LOCS III score was 3.63 ± 0.92, the mean DLI was 2.95 ± 1.30 and the mean PNS was 2.36 ± 1.20. The acquisition success rates for the Anterion®, Argos®, IOLMaster® 700, Pentacam® AXL and OcuScan® RxP biometers were 94.12%, 100%, 98.04%, 60.78% and 100%, respectively. There were significant differences in the success rates between biometers (*P* = 0.014). There were statistically significant differences between biometers for all parameters evaluated (*P* < 0.05). The range of the limit of agreement (LoA) for all comparisons of K1 and K2 were > 1.00 D. The LoA for WTW ranged from 0.095 to 1.050 mm. The LoA for ACD and LT ranged from 0.307 to 0.114 mm and from 0.378 to 0.108 mm, respectively. The LoA for axial length ranged from 0.129 to 2.378 mm.

**Conclusions:**

Among optical biometers, those based on SS-OCT technology are more successful at measuring axial length in eyes with dense cataracts.

*Trial Registration:* The study was registered with the National Institutes of Health (clinical trial identifier NCT05239715, http://www.clinicaltrials.gov).

**Supplementary Information:**

The online version contains supplementary material available at 10.1186/s40662-023-00352-3.

## Background

Continual technological developments in optical biometry have allowed clinicians to obtain axial length measurements even in dense cataracts. A decline in failure rates and more complete, accurate measurements of ocular dimensions has led to improved refractive and visual outcomes for patients. This development has led to more predictable cataract surgery with excellent outcomes. From the early application of ultrasound [1], to the latest noninvasive optical techniques for measuring axial distances in the eye, many studies have reported on the viability of these techniques when calculating the intraocular lens (IOL) power.

The main limitation of optical biometry is cataract density, as biometry techniques cannot obtain enough reflected light from the retina. Inaccurate measurements and high variations in axial length are frequently encountered when examining eyes with dense cataracts. It has been reported that partial coherence interferometry (PCI)-based biometers fail to acquire accurate measurements of the axial length in about 15% of cataract patients (8%–21%) [2]. Failure was mainly due to posterior subcapsular cataracts (PSC) and mature cataracts and a Lens Opacities Classification System (LOCS) III [3] scale value of 3.5 was suggested as the clinical cut-off for the use of PCI biometers [4]. To improve postoperative refractive outcomes, some authors suggested that presurgical biometry suites should use both PCI and ultrasound techniques, relying on ultrasound biometry alone for those cases in which PCI measurements are unfeasible [4]. Improved software has recently enhanced the signal-to-noise ratio, so valid measurements can now be made in eyes where PCI had previously failed (especially in eyes with PSC), more than halving the failure rate from 10% to 4.7% [2]. Axial length measurement using optical low coherence reflectometry (OLCR) was impossible in 10% of eyes, correlating significantly with the presence of PSC of LOCS III grade ≥ 4 [5]. A specific study reporting axial length measurements with PCI and OLCR found failure rates of about 35%–38%, with increased severity of PSC proving to be a problem for both types of devices [6]. However, a new axial measurement mode using OLCR in eyes with a dense cataract significantly reduced the failure rate to 1.6% [7]. More recently, swept-source optical coherence tomography (SS-OCT) has been applied to optical biometry. This new technology has several advantages over other technologies and SS-OCT biometers are likely to become the gold standard for ocular biometry [8]. A recent publication concluded that SS-OCT biometers showed the best agreement for axial distances compared to other optical biometers on the market [9]. The main difference between SS-OCT and PCI is that the former uses Fourier-domain OCT, which enables better penetration and may help improve the success rate for axial length measurement in eyes with severe crystalline lens opacity [10]. Different ocular measurements using these optical biometers may have led to significantly different IOL predictions and should be judged clinically [11]. A recent review focused on the outcomes reported when SS-OCT biometry failed during axial length measurement [12]. The study indicated that SS-OCT biometers produced only low failure rates when measuring axial length. The authors highlighted that in the few cases where the measurement was impossible, most cataracts were mature white or LOCS III grade ≥ 4. They concluded that SS-OCT biometry surpasses other optical technologies and may be considered the gold standard for measuring the axial length in any type of cataract. Few studies have specifically assessed exactly how effective SS-OCT technology is in eyes with dense cataracts [13–18].

It should be borne in mind that different studies use different definitions for dense/mature/advanced cataract, specifically when subjective classifications are used. As a result, the grade of cataract among patients included in those studies varies, which may have a large influence on the acquisition success rates and therefore seriously complicates any direct comparisons [12]. In such cases we believe that objective metrics, such as the dysfunctional lens index (DLI) [19–22] and Pentacam® nucleus staging (PNS) [23–27], should be considered in any thorough assessment of acquisition success rate and the agreement between biometers.

Therefore, considering it is usually difficult to accurately measure axial length in eyes with dense cataracts and due to the lack of comprehensive comparisons of SS-OCT biometers using objective metrics in eyes with different degrees of cataract, the aim of this study was to assess the axial length acquisition success rates and to compare the measurements of keratometry (K1: flattest keratometry; K2: steepest keratometry), white-to-white distance (WTW), anterior chamber depth (ACD), lens thickness (LT) and axial length in eyes with dense cataracts obtained with the Anterion® SS-OCT (Heidelberg Engineering, Heidelberg, Germany), Argos® SS-OCT (Alcon Laboratories, Fort Worth, USA) and IOLMaster® 700 SS-OCT (Carl Zeiss Meditec, Jena, Germany) biometers. We also compared measurements obtained with the Pentacam® AXL PCI (Oculus, Wetzlar, Germany) biometer and the OcuScan® RxP (Alcon Laboratories, Fort Worth, TX, USA) ultrasound biometer, and collected DLI and PNS objective metrics related to the degree of the cataract.

## Methods

This single-center, prospective, observational study was conducted in accordance with the tenets of the Declaration of Helsinki and approved by the Ethics Committee of the Hospital Clínico San Carlos (Madrid, Spain, Reference 21/599-O_P) and the Valencia regional committee on postmarketing studies CAEPRO (Valencia, Spain). All patients provided written informed consent before they were enrolled in the study. The study was registered with the National Institutes of Health (clinical trial identifier NCT05239715, http://www.clinicaltrials.gov).

### Biometers

The Anterion® SS-OCT operates at a wavelength of 1300 nm and 50,000 scans/s to obtain a 2D OCT scan of the eye. Anterior and posterior corneal curvatures were measured using 65 B-scans in the 3 mm zone considering refractive indices of 1.3375 and 1.376, respectively (only anterior K readings were considered in this study). The Argos® SS-OCT operates at a wavelength of 1060 nm and a bandwidth of 20 nm to obtain a 2D OCT scan of the eye using 3000 A-scans/s. K was measured from the OCT image using a 2.2 mm diameter ring made up of 16 LEDs and a corneal refractive index of 1.3375. The biometric parameters were measured from the OCT scan, considering different refractive indices: 1.376 for the cornea, 1.336 for the aqueous and vitreous humors, and 1.410 for the lens.

The Argos® biometer has an enhanced retinal visualization (ERV) mode in which the optical path length is measured by minimizing the effect of attenuation and by changing the OCT sensitive position to the retinal side. By combining this optical path length with the anterior segment information up to the posterior surface of the crystalline lens, measured with the standard mode, the axial length in ERV mode is calculated. The IOLMaster® 700 SS-OCT uses a wavelength of 1,055 nm (varying from 1035 to 1095 nm) at a rate of 2000 scans/s to obtain a 2D OCT image of the eye. K was measured using a telecentric K projecting the 950 nm light source onto three zones of the cornea (1.5, 2.5 and 3.2 mm in a mean corneal radius of 7.9 mm) at a refractive index of 1.3375. The Pentacam® AXL is based on the Scheimpflug principle and has an additional module that measures axial length with PCI. Its light source is a blue LED that emits at a wavelength of 475 nm. Corneal images were captured by a rotating Scheimpflug high-definition camera that provided anterior K values using a reference surface. This biometer does not measure LT. The OcuScan® RxP is a contact ultrasound biometer that provides an A-scan biometry.

### Patients and procedure

All patients underwent a full eye examination, including preoperative corrected distance visual acuity (CDVA), subjective refraction, and slit-lamp and dilated fundus examinations. The inclusion criteria were eyes with a LOCS III grade of ≥ 3 for nuclear, cortical or PSC, and a DLI of ≤ 5, as determined with the iTrace® device (Tracey Technologies, Houston, TX, USA). The LOCS III is the current validated gold standard for slit-lamp assessments of cataracts [3]. The DLI is an objective lens performance metric derived from internal higher-order aberrations, pupil size and contrast sensitivity data that has been used to assess cataracts (on a scale of 0, very poor, to 10, excellent) [19–22]. Lens density was also evaluated using the Pentacam® PNS value, which returns average and maximum lens densities with a cataract grading score (from 0 to 5) and has been widely used for this purpose in the literature [23–27]. Exclusion criteria considered ocular trauma, previous refractive, glaucoma, vitreoretinal or IOL surgeries, previous extended contact lens wear, poor fixation and/or corneal disease (e.g., keratoconus).

K1, K2, WTW, ACD, LT and axial length parameters were measured using the three optical biometers in a random order during a morning session. The axial length could not be measured using the Argos® biometer in the standard mode, so it was measured using the ERV mode. We measured the axial length at least three times. Ultrasound biometry using anesthetic drops was performed after all other measurements to avoid any changes to the cornea caused by the ultrasound probe. Only one eye from each patient was used for the data analysis (in cases where both eyes could be included the eye considered was chosen randomly) and all the devices were calibrated prior to each measurement session.

IOL power was calculated using the Barrett True-K formula with emmetropia (nearest negative number to zero) as the target for the IOL. Snellen decimal CDVA and uncorrected distance visual acuity (UDVA), and subjective refraction were measured with a mean follow-up of 3 months post-surgery.

### Sample size calculation

Axial length acquisition success rates of 89.9% (Argos®) and 63.6% (IOLMaster® 700) in advanced cataracts are known from a previous study [16]. The sample size was computed using McNemar’s test for correlated proportions, requiring the inclusion of the proportions of discordant pairs. We have considered a type I error of 5%, two-tailed hypothesis, 90% power, P10 = 26% (the frequency at which subjects could be measured with the Argos® but not with the IOLMaster® 700), P01 = 1% (the frequency at which subjects could not be measured with the Argos® but they could with the IOLMaster® 700). This gave a sample size of 42, and with an estimated drop-out rate of 10%, we decided to recruit a total of 50 eyes.

### Statistical analysis

Axial length acquisition success rates were calculated as percentages and other variables as the mean, standard deviation (SD), and minimum and maximum values. χ^2^ test was used to compare acquisition success rates between the five arms. With respect to the other variables (K, WTW, ACD, LT and axial length), the normality of the distribution was checked using the Shapiro–Wilk test. Statistically significant differences between the measurements for the five arms were assessed with repeated measures analysis of variance (rANOVA). The Tukey test was used for post-hoc analysis to compare the data between arms whenever rANOVA revealed significant differences between measurements. This test gave us the significance level for paired differences between the individual conditions. Statistical significance was set at a *P* value of less than 0.05. In addition, the agreement between the biometers was studied by applying a Bland–Altman analysis. We also determined the average difference, the confidence interval of the average difference at 95%, and the 95% limits of agreement (LOA, calculated as the mean difference ± 1.96 SD).

## Results

In total, we examined 51 eyes of 51 patients (27 females and 24 males) with dense cataracts. The mean and SD of the DLI measured with the iTrace® device was 2.95 ± 1.30 (rang 0.17–4.91), the PNS measured with the Pentacam® device was 2.36 ± 1.20 (rang 1–5). The mean LOCS III for the study sample was 3.63 ± 0.92, ranging from 3 to 6. There were 30 (58.82%), 14 (27.45%), 3 (5.88%) and 4 eyes (7.84%) with grades 3, 4, 5 and 6, respectively, among the subjects. The patients have a mean age of 72.06 ± 8.45 years (50–89 years). We only studied one eye in each subject, and all eyes were measured using the five biometers. No safety events as adverse events, serious adverse events or adverse device deficiencies were reported during the whole duration of the study. The mean spherical equivalent of the eyes in our sample was − 2.07 ± 4.31 D (mean ± SD), with a range of − 15.75 to 3.63 D, and the CDVA was 0.40 ± 0.23 logMAR. Table 1 shows the mean values, SD, and ranges for the different parameters obtained using the five devices. Note that LT was not obtained from the Pentacam® AXL and the axial length was only considered when its measurement was possible. There were statistically significant differences between devices in results for K1, K2, WTW, ACD and axial length (*P* < 0.05). Mean CDVA, UDVA and spherical equivalent post-surgery were 0.95 ± 0.14, 0.81 ± 0.23 and − 0.15 ± 0.48 D, respectively.

**Table 1:** Mean ± standard deviation (range) [95% confidence interval] of different parameters for the five devices

Parameter	Anterion	Argos	IOLMaster 700	Pentacam AXL	OcuScan RxP	*P* value
K1 (D)	43.45 ± 1.40 (39.83–46.08) [43.07–43.84] n = 51	43.53 ± 1.39 (39.76–46.15) [43.15–43.91] n = 51	43.49 ± 1.41 (39.84–46.58) [43.10–43.88] n = 51	43.38 ± 1.29 (39.60–46.00) [43.03–43.73] n = 51	NA	0.005*
K2 (D)	44.43 ± 1.39 (40.48–48.10) [44.05–44.81] n = 51	44.60 ± 1.35 (40.55–48.20) [44.23–44.97] n = 51	44.51 ± 1.35 (40.55–48.01) [44.14–44.88] n = 51	44.38 ± 1.32 (40.30–48.20) [44.02–44.74] n = 51	NA	< 0.001*
WTW (mm)	11.67 ± 0.40 (10.40–12.49) [11.56–11.78] n = 51	11.85 ± 0.37 (11.10–12.59) [11.75–11.95] n = 51	11.87 ± 0.40 (10.60–12.60) [11.76–11.98] n = 51	11.79 ± 0.38 (10.50–12.60) [11.68–11.89] n = 48	NA	< 0.001*
ACD (mm)	3.22 ± 0.37 (2.41–4.16) [3.12–3.33] n = 51	3.28 ± 0.37 (2.52–4.13) [3.17–3.38] n = 51	3.14 ± 0.37 (2.31–4.02) [3.04–3.24] n = 51	3.20 ± 0.38 (2.40–4.07) [3.09–3.30] n = 51	NA	< 0.001*
LT (mm)	4.59 ± 0.37 (3.61–5.29) [4.49–4.70] n = 47	4.60 ± 0.42 (3.56–5.93) [4.48–4.72] n = 51	4.58 ± 0.41 (3.62–5.94) [4.46–4.69] n = 51	NA	NA	< 0.001*
AL (mm)	23.77 ± 1.56 (20.60–28.51) [23.33–24.21] n = 48	23.94 ± 1.97 (20.67–33.22) [23.40–24.48] n = 51	23.98 ± 2.06 (20.64–33.70) [23.41–24.55] n = 50	23.95 ± 1.74 (22.16–29.33) [23.34–24.56] n = 31	23.78 ± 1.98 (19.86–33.01) [23.23–24.32] n = 51	< 0.001*

*K* = keratometry; *K1* = flattest keratometry; *K2* = steepest keratometry; *WTW* = white-to-white distance; *ACD* = anterior chamber depth; *LT* = lens thickness; *AL* = axial length; *NA* = not available; *n* = number of eyes in which measurement was obtained

^*^Significant differences < 0.05 (rANOVA test)

### Axial length acquisition success rates

**Fig. 1:**
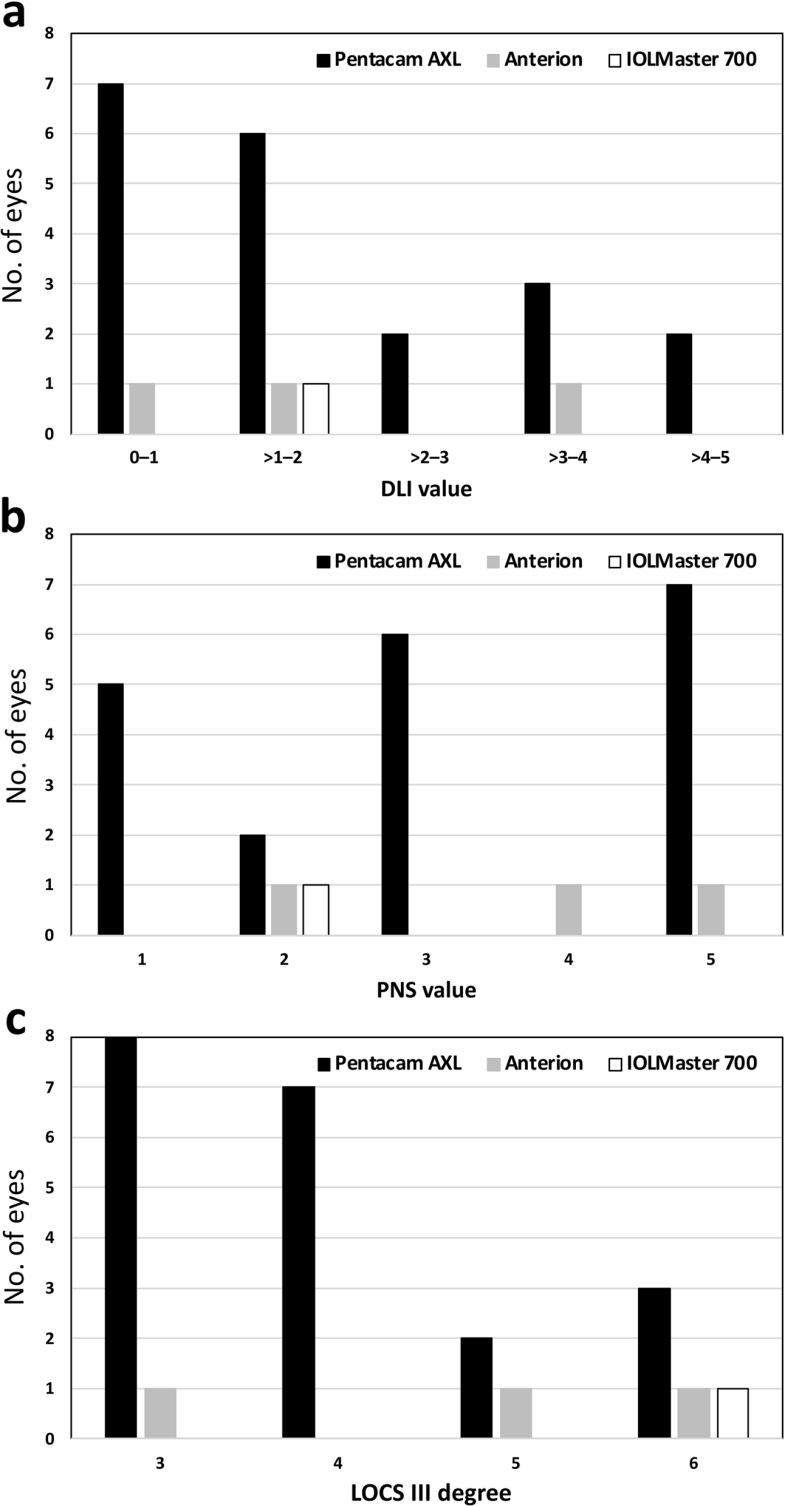
Distribution of eyes in which the axial length could not be measured as a function of the DLI (**a**), PNS (**b**) and LOCS III score (**c**) for the Pentacam® AXL, Anterion® and IOLMaster® 700 optical biometers. Note that when the iTrace® could not return a result, the DLI value was considered to be 0 (2 eyes) and the Pentacam was taken as 5 (3 eyes). DLI, dysfunctional lens index; LOCS III, Lens Opacities Classification System III; PNS, Pentacam® nucleus staging

The axial length acquisition success rates were 94.12% (48 eyes), 100% (51 eyes), 98.04% (50 eyes), 60.78% (31 eyes) and 100% (51 eyes) for the Anterion®, Argos®, IOLMaster® 700, Pentacam® AXL and OcuScan® RxP biometers, respectively. The χ^2^ test revealed that there was a statistically significant difference between these percentages (*P* = 0.014). Figure 1 shows the distribution of eyes in which the axial length could not be measured with the biometers as a function of either the objective DLI and PNS values obtained with the iTrace® and Pentacam® biometers, respectively, or the subjective LOCS III scale. In just one eye, axial length could not be measured with the Anterion®, IOLMaster® 700 or Pentacam® AXL devices, but it was possible using the Argos® in ERV mode and the OcuScan® RxP. In the three cases that the Argos® was unable to determine axial length in standard mode, the parameter was successfully measured in all subjects using the ERV mode.

### Agreement between biometers

**Fig. 2:**
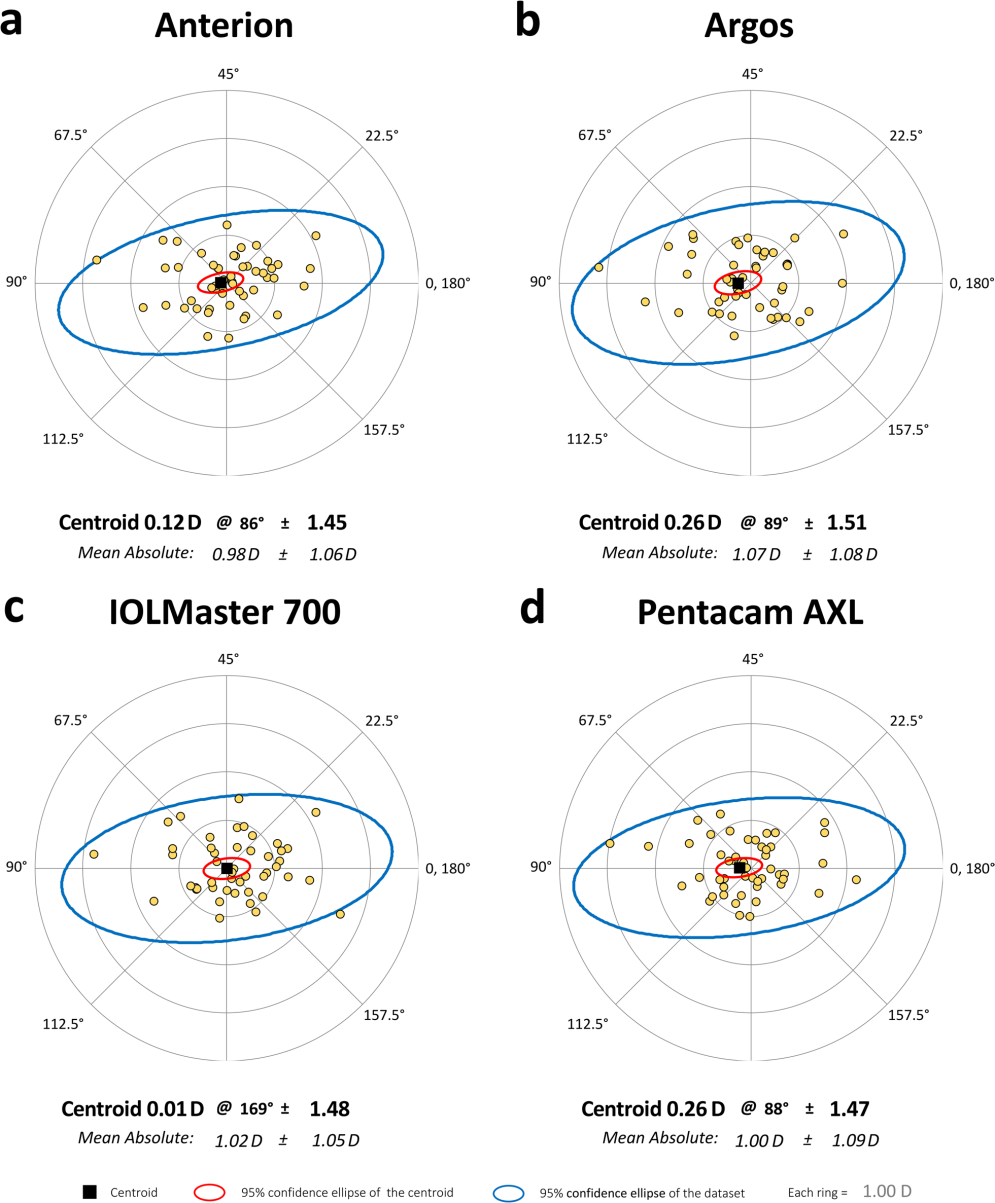
Preoperative corneal astigmatism using double-angle plot for Anterion (**a**), Argos (**b**), IOLMaster 700 (**c**) and Pentacam AXL (**d**) optical biometers evaluated (n = 51). These graphs show centroid values, the standard deviation and 95% confidence ellipses (each ring = 1.00 D)

Table 2 shows the level of agreement between the five biometers, with the mean difference ± SD and 95% LoA for all pairwise comparisons between biometers. Note that there were three possible comparisons for LT, six for K1, K2, WTW and ACD, and 10 for axial length according to the values obtained using each biometer. The rANOVA analysis revealed statistically significant differences between the five biometers for all the parameters evaluated (Table 1, *P* < 0.05), while the *P* values from the post-hoc Tukey test are shown in Table 2. There were statistically significant inter-device differences for all ACD and LT comparisons, and some for K1, K2, WTW and axial length (see specific *P* values in Table 2, *P* < 0.05). Bland–Altman plots are also included in several figures to highlight the inter-device differences for K1 and K2 (Additional file 1: Fig. S1), WTW (Additional file 2: Fig. S2), ACD (Additional file 3: Fig. S3), LT (Additional file 4: Fig. S4) and axial length (Additional file 5: Fig. S5). In addition, the preoperative astigmatism obtained with the four optical biometers is shown in a double-angle plot (Fig. 2) [28].

**Table 2:** Agreement between the devices for the parameters keratometry (K), white-to-white distance (WTW), anterior chamber depth (ACD), lens thickness (LT) and axial length

Parameter/device	Mean difference ± SD	95% LoA	*P* value
*K1 (D)*			
Anterion vs. Argos	− 0.084 ± 0.294	− 0.660, 0.493	0.176
Anterion vs. IOLMaster 700	− 0.038 ± 0.260	− 0.548, 0.472	0.512
Anterion vs. Pentacam AXL	0.072 ± 0.455	− 0.821, 0.965	0.585
Argos vs. IOLMaster 700	0.046 ± 0.257	− 0.458, 0.549	0.915
Argos vs. Pentacam AXL	0.156 ± 0.395	− 0.618, 0.929	0.005*
IOLMaster 700 vs. Pentacam AXL	0.110 ± 0.446	− 0.764, 0.984	0.041*
*K2 (D)*			
Anterion vs. Argos	− 0.166 ± 0.341	− 0.836, 0.504	0.001*
Anterion vs. IOLMaster 700	− 0.084 ± 0.266	− 0.606, 0.438	0.272
Anterion vs. Pentacam AXL	0.040 ± 0.409	− 0.762, 0.841	0.794
Argos vs. IOLMaster 700	0.082 ± 0.302	− 0.509, 0.673	0.221
Argos vs. Pentacam AXL	0.206 ± 0.350	− 0.480, 0.892	< 0.001*
IOLMaster 700 vs. Pentacam AXL	0.124 ± 0.386	− 0.634, 0.881	0.033*
*WTW (mm)*			
Anterion vs. Argos	− 0.191 ± 0.264	− 0.709, 0.326	< 0.001*
Anterion vs. IOLMaster 700	− 0.202 ± 0.189	− 0.574, 0.170	< 0.001*
Anterion vs. Pentacam AXL	− 0.111 ± 0.166	− 0.436, 0.213	< 0.001*
Argos vs. IOLMaster 700	− 0.010 ± 0.262	− 0.524, 0.503	0.168
Argos vs. Pentacam AXL	0.080 ± 0.267	− 0.444, 0.605	0.972
IOLMaster 700 vs. Pentacam AXL	0.083 ± 0.177	− 0.264, 0.430	0.061
*ACD (mm)*			
Anterion vs. Argos	− 0.052 ± 0.076	− 0.202,0.098	0.006*
Anterion vs. IOLMaster 700	0.081 ± 0.029	0.024,0.138	< 0.001*
Anterion vs. Pentacam AXL	0.026 ± 0.068	− 0.107,0.159	0.004*
Argos vs. IOLMaster 700	0.133 ± 0.078	− 0.021,0.286	< 0.001*
Argos vs. Pentacam AXL	0.078 ± 0.043	− 0.007,0.163	< 0.001*
IOLMaster 700 vs. Pentacam AXL	− 0.055 ± 0.066	− 0.183,0.074	< 0.001*
*LT (mm)*			
Anterio vs. Argos	0.031 ± 0.075	− 0.115,0.178	< 0.001*
Anterion vs. IOLMaster 700	0.048 ± 0.027	− 0.006,0.102	< 0.001*
Argos vs. IOLMaster 700	0.023 ± 0.096	− 0.166,0.211	0.005*
*Axial length (mm)*			
Anterion vs. Argos	0.013 ± 0.066	− 0.116,0.142	0.998
Anterion vs. IOLMaster 700	− 0.006 ± 0.033	− 0.071,0.059	0.493
Anterion vs. Pentacam AXL	− 0.026 ± 0.181	− 0.381,0.330	0.393
Anterion vs. OcuScan RxP	0.146 ± 0.528	− 0.888,1.181	0.148
Argos vs. IOLMaster 700	− 0.028 ± 0.087	− 0.199,0.143	0.701
Argos vs. Pentacam AXL	− 0.044 ± 0.204	− 0.444,0.355	0.598
Argos vs. OcuScan RxP	0.164 ± 0.515	− 0.845,1.173	0.068
IOLMaster 700 vs. Pentacam AXL	− 0.023 ± 0.200	− 0.415,0.369	1.000
IOLMaster 700 vs. OcuScan RxP	0.180 ± 0.523	− 0.845,1.205	< 0.001*
Pentacam AXL vs. OcuScan RxP	0.234 ± 0.607	− 0.950,1.428	< 0.001*

^*^Significant differences < 0.05 (Tukey’s test)

## Discussion

A leading challenge in optical biometry is the axial measurement of eyes with dense cataracts. PSC and mature cataracts commonly cause measurement acquisition failure. It has been reported that failure rates vary significantly depending on the optical technology used to measure axial length, with SS-OCT-based biometers producing the lowest failure rates [12]. As mentioned earlier, few studies have specifically analyzed the technology’s degree of success in eyes with dense cataracts [13–18]. The main purpose of this study was to evaluate acquisition success rates using different SS-OCT-based biometers in eyes with dense cataracts. To the best of our knowledge, there have been no studies that evaluated these optical biometers using objective metrics for describing cataract density.

Our results revealed statistically significant differences in the axial length acquisition success rates between biometers (*P* = 0.014). The acquisition rates of the SS-OCT biometers [Argos® (100%), IOLMaster® 700 (98.04%) and Anterior® (94.12%)] were significantly higher than the PCI biometer [Pentacam® AXL (60.78%)]. Additional file 6: Table S1 shows the axial length acquisition success rates obtained in eyes with dense cataracts from different clinical studies using optical biometers based on PCI, OLCR and SS-OCT technologies. It shows the cataract type for each study sample as well as the type of cataract that could not be measured. Hirnschall et al. [13] evaluated the use of the IOLMaster® 700 SS-OCT for eyes in which the axial length could not be measured using the IOLMaster® 500 PCI biometer. The authors looked at 23 subjects with failed acquisition using the IOLMaster® 500 and only two could not be measured with the IOLMaster® 700 SS-OCT. The 2 patients whose axial length could not be measured with the IOLMaster® 700 had nuclear cataracts. The study reported an acquisition success rate of 91.3%, and our value was 98.04%. The authors concluded that SS-OCT technology significantly improves the rate of attainable axial eye length measurements, especially in eyes with PSC, but also in eyes with dense nuclear cataracts, except for white cataracts. The main reason for SS-OCT’s greater success rate is the use of a longer wavelength (1,055 nm) compared to PCI technology (780 nm), as shorter wavelengths mean shallower penetration due to scattering [29]. Henriquez et al. [14] studied 45 eyes with cataracts of grade ≥ 3 (using LOCS III) for nuclear color (NC) and nuclear opalescence (NO), PSC and/or cortical cataract. They used the IOLMaster® 700 SS-OCT, Galilei G6 OLCR and Pentacam® AXL PCI biometers and assessed lens density by means of the LOCS III and PNS metric. The mean and SD for the PNS score in their sample was 3.21 ± 1.33. After three attempts, the Pentacam® AXL, Galilei G6 and IOLMaster® 700 could measure axial length in 37.7%, 42.2%, and 84.4% of the eyes, respectively. These authors reported the mean cataract density based on LOCS III for those eyes that could not be measured using the three devices (see Additional file 1: Table S1 for details). Note that these three devices use different wavelengths—1055 nm (IOLMaster® 700), 880 nm (Galilei G6) and 474 nm (Pentacam® AXL)—and higher wavelengths produce a better signal-to-noise ratio hence improving tissue penetration (but higher signal-to-noise ratio is also achieved due to different detection schemes used in those devices and different types of detector technologies). Additionally, wavelength correlates with the acquisition success rate. Our acquisition rates were higher for the IOLMaster® 700 and Pentacam® AXL. Note that the mean LOCS III and PNS scores were higher than those for our sample when axial length could not be measured (specifically, for the Pentacam® AXL biometer, the values were 4.00 ± 1.08 and 2.76 ± 1.52, respectively). Vasavada et al. [15] compared the Lenstar LS900 OLCR and the Tomey OA-2000 SS-OCT and found that the LS900 was unable to measure axial length in 22.58% eyes, whereas the Tomey OA-2000 failed in only in 1.6% eyes. Note that the study sample included eyes with low-grade cataracts. The authors concluded that axial length measurement on OCLR failed in one-fifth of the subjects with dense cataracts, which consist of white mature, dense PSC with a posterior capsule plaque or posterior polar cataracts.

Tamaoki et al. [16] assessed the Argos®, IOLMaster® 700 and OA-2000 SS-OCT biometers reporting acquisition rates of 89.9%, 63.6% and 80.8%, respectively. The IOLMaster® 700 had a significantly lower acquisition rate than that of the Argos® (*P* < 0.0001) and OA-2000 (*P* = 0.011) biometers, but there was no statistically significant difference between the Argos® and OA-2000 in this regard. The cataract type that resulted in failed acquisitions were mainly mature or white cataracts (see Additional file 1: Table S1). Data could not be acquired for three eyes with grade 4 nuclear sclerotic cataract accompanied by PSC using the IOLMaster® 700. However, acquisition was successful with the other two biometers. As there were only six cases of grade ≥ 4 nuclear sclerotic cataracts accompanied by PSC in their study, the authors suggested that further investigation is needed to elucidate how this condition may affect axial length measurements. The same authors [17], compared the IOLMaster® 700 and Argos® SS-OCT biometers in eyes with a nuclear hardness ≥ 4 [30, 31]. If the Argos® biometer failed to measure the axial length using the standard mode, it was measured using the ERV mode. They did not observe any statistically significant difference between the acquisition success rates for the Argos® in standard mode (69.5%) and the IOLMaster® 700 (61.5%; *P* = 0.083). However, they found that the overall acquisition success rate of the Argos® standard and ERV modes combined (93.4%) was significantly higher than that of the IOLMaster® 700 (*P* < 0.001). Among the 82 eyes that could not be measured using the IOLMaster® 700, 25 eyes had a white cataract. Of these 25, the Argos® successfully measured the axial length of 16.0% in standard mode and 60.0% in ERV mode. The percentages obtained in our study were higher than those reported by Tamaoki et al. [16, 17]. In another study, González-Godínez et al. [18] used the IOLMaster® 700 and IOLMaster® 500 and reported a failure rate of 68.57% and 21.43%, respectively (*P* = 0.007). The analysis revealed that PCI acquisition success rates were 69.23% of NO4, 66.6% of P3 and 15.3% of mixed cataracts, while for SS‑OCT they were 100% of NO4, NO5, P3 and P5 and 76.9% of mixed cataracts. The IOLMaster® 700 biometer had rates of 100% of NO4, NO5 and P3, and 88.8% of P4. They reported failure rates for a mixed cataract group (composed of PSC P > 3 and cortical C ≥ 4 or nuclear opalescence NO ≥ 4) of 100% when employing the PCI biometer and 40% for the SS‑OCT. The authors suggested that the cut‑off for the SS-OCT biometer may well be up to P4 and NO5. As for dense nuclear opacity above NO5 and intumescent cataracts, immersion ultrasound biometers remain the best option.

As highlighted by Tamaoki et al. [17], the difficulty of measuring axial length in white cataracts is due to light scattering even when using SS-OCT biometers operating at long wavelengths (see failures for SS-OCT as a function of LOCS III score in our study, Fig. 1, bottom panel). Tamaoki et al. [17] reported that the axial length of 48.6% of white cataracts could not be measured using the Argos® in standard mode but acquisition was successful in the ERV mode. In our study, axial length could not be measured with the Argos® in standard mode for three eyes with grade 6 LOCS III scores, but all of them were successfully measured with the ERV mode. OCT sensitivity decreases with depth and particularly in the case of dense cataracts. This precludes retinal pigment epithelium layer detection and vitreous length measurement due to light energy attenuation. In ERV mode, optical path length is measured using the same principle as that used in enhanced depth imaging for choroidal imaging [17, 32]. We believe this mode is an excellent option for measuring axial length in complicated cases. Other authors [33] have recently concluded that pharmacologic pupil dilation improved the quality of the IOLMaster® 700’s biometrically measured axial length in patients with low-quality measurement due to dense cataract. High-quality axial length measurements were successfully obtained in 60 of the 79 eyes (75.95%) following pupil dilation in this study. The mean standard deviation of the measurements obtained decreased significantly (*P* < 0.001) and the mean difference before and after pupil dilation was 0.03 ± 0.07 mm (*P* < 0.001).

Regarding the agreement between devices for the different parameters, our results revealed statistically significant differences between the five biometers (Table 1, *P* < 0.05). The post-hoc Tukey test showed that statistical significance depended on which pairwise comparisons were analyzed between biometers and which parameter was being examined. Specifically, significant differences were found between devices for all ACD and LT comparisons, and some for K1, K2, WTW and axial length (Table 2, *P* < 0.05).

Results for K showed that the minimum mean differences were obtained for the comparison between the Anterion® vs. IOLMaster® 700 (− 0.038 D) for K1 and between the Anterion® vs. Pentacam® AXL (0.040 D) for K2, but neither case was statistically significant (*P* > 0.05). The maximum mean differences were between the Argos® vs. Pentacam®, with both K1 (0.156 D) and K2 (0.206 D) returning a statistically significant difference (*P* < 0.05). It has been suggested that a difference of 1.00 D in the K value would cause a difference of about 1.40 D in the IOL power calculation [35]. If we used the K values to calculate the IOL power and took 0.206 D as the maximum mean difference, then it would lead to a difference of about 0.28 D in the IOL power. The small differences in agreement reported for all K measurement comparisons led to clinically insignificant changes in the IOL power calculation because of the 0.50 D step in IOL manufacturing. Nevertheless, it is worth noting that the range of LoA varied as a function of the comparison and all comparisons were > 1.00 D, which is broad enough to produce a significant change in IOL power (see Table 2 and Additional file 1: Fig. S1). Others have reported that K values cannot be interchanged between the Anterion®, IOLMaster® 700 and Pentacam® AXL biometers [10]. The mean differences reported for WTW measurements varied from − 0.01 mm for the Argos® vs. the IOLMaster® 700 to − 0.20 mm between the Anterion and IOLMaster® 700 (Table 2). The LoA range was ≥ 0.60 mm, which may be clinically significant. For IOL power, we did observe differences when using the Holladay 2 and Barrett formulas with WTW as a variable. WTW measurements between these devices cannot be considered interchangeable. Tañá-Rivero et al. concluded that WTW data may be considered interchangeable between the Anterion® and IOLMaster® 700, and the Anterion® and Pentacam® HR but not between the IOLMaster® 700 and Pentacam® HR [36], and that the IOLMaster® 700 measured the largest WTW distances and the Pentacam® AXL the shortest [10]. As for ACD, we found statistically significant differences between all pairwise comparisons, although they were very small (about 0.1 mm). A previous study comparing the Anterion® and Pentacam® HR biometers reported similar mean differences [39] and the Anterion®, IOLMaster® 700 and Pentacam® AXL can be used interchangeably [10]. On average, it has been reported that a 1 mm deviation in ACD could lead to a refractive error of 1.50 D in IOL power [34] (maximum LoA was about 0.30 D, resulting in a change in IOL power of less than 0.50 D). Therefore, we can conclude that the ACD differences reported between biometers will not affect IOL power calculation. Mean differences for LT were less than 0.05 mm with a maximum LoA range of 0.378 mm. Note that a 0.2 mm increase in LT would change the IOL power by 0.20 D. However, taking into account our mean differences, this may not have a clinically significant impact on the IOL power calculation when using the Olsen or Holladay 2 formulas [37, 38]. We believe the three devices can be used interchangeably for LT measurements, as has been reported previously for comparisons of SS-OCT biometers [10, 40]. Our mean differences obtained for axial lengths between optical biometers were all less than 0.1 mm, except when compared to the OcuScan® RxP which returned higher values (up to 0.234 mm for the comparison between Pentacam® AXL and OcuScan® RxP; see Table 2). If we consider that a 0.1 mm error in axial length would yield a refraction error of about 0.27 D [34], then the differences between optical biometers would not affect the IOL power calculation but would affect any comparisons with ultrasound. Hence, we believe that only optical biometers can be used interchangeably (as previously reported with SS-OCT and PCI [10] in non-dense cataracts and healthy eyes [9]). However, the LoA ranges reach high values, surpassing the limits considered clinically negligible, and this must be taken into account in the IOL power calculation.

Only Henriquez et al. [14] has compared the IOLMaster® 700 and Pentacam® AXL in 45 eyes with mature cataracts. They reported significant inter-device differences for axial length and K2 (*P* = 0.012 and 0.034, respectively), but not for K1 and ACD (*P* > 0.1). The absolute mean difference was 0.05 mm for axial length and 0.33 D for K2. Our results are distinct because we have found statistically significant inter-device differences for all parameters (K1, K2, WTW and ACD), but not for axial length (*P* > 0.99, with a mean difference of − 0.023 mm and a LoA range of 0.784 mm; see Table 2). As discussed previously, the mean LOCS III and PNS scores were higher than those observed for our sample where the measurement was not possible, which could be a source of differences between the studies. Tamaoki et al. [17] compared pre- and postoperative axial length measurements and found mean absolute errors of 0.05, 0.10, 0.08 and 0.12 mm for the Argos®, IOLMaster® 700, Argos® in ERV mode and ultrasound biometers, respectively. The absolute difference for the Argos® was significantly lower than for the IOLMaster® 700, Argos® in ERV mode and ultrasound biometers (*P* < 0.001, *P* = 0.008 and *P* < 0.001, respectively). Comparing the Argos® with and without ERV, they indicated that despite being higher than the difference obtained with the standard mode, this value can be considered clinically negligible. They also found an absolute error of 0.12 mm using the ultrasound biometer, almost the same as that observed with the ERV mode. They suggested that measurements with the ultrasound biometer are also important since 22% of cases showed an axial length error ≥ 0.2 mm. It should be noted that for pre- and post-surgical measurements, it helps to consider the LoA when performing the Bland–Altman analysis [11] and, secondly, the Argos® measures axial length using segmental refractive indices and the components of the eye are different in this comparison (with more variability if different IOLs are used, as indicated by Tamaoki et al. [17]). Gonzalez-Godinez et al. [18] compared the IOLMaster® 700 and an ultrasound biometer and found a poor level of agreement: the IOLMaster® 700 mean axial length was 0.15 mm shorter than the ultrasound biometer (*P* = 0.005) with an LoA of 1.56 mm. Our values, when compared to another ultrasound biometer, were slightly higher: 0.180 mm and 2.049 mm for the mean difference and LoA, respectively. Differences between techniques (including which retinal layer is measured, velocity and refractive index changes in dense cataracts when using ultrasound biometry) may explain the poor agreement. A recent network-based big data analysis demonstrated that when considering the measurement of axial length, contact ultrasound biometry obtains lower values compared with optical biometers [41].

## Conclusion

This study assessed axial length acquisition success rates in eyes with dense cataracts using different optical biometers and while considering objective metrics for describing cataract density. Our results show that the LoA obtained for each comparison should be assessed carefully to consider their interchangeability, assuming measurements can be obtained. In some eyes with dense cataracts, the axial length cannot be measured using optical biometry and so ultrasound biometry is required. SS-OCT biometry increases the acquisition rate, especially when operating in ERV mode, and so it provides the most reliable measurements for use in IOL power calculations.

## Supplementary Information

**Additional file 1: Fig. S1.** Bland–Altman plots of the mean difference versus the average of K1 (flattest keratometry, a to f) and K2 (steepest keratometry, g to l) used to compare the different devices. The plots show the mean (continuous line), lower and upper limits of agreement (± 1.96 SD [standard deviation], peripheral dotted lines), and the lower and upper confidence intervals (95%). The *P* values are included in each comparison (*significant differences < 0.05).

**Additional file 2: Fig. S2.** Bland–Altman plots of the mean difference versus the average of white-to-white (WTW) distance used to compare the different devices: Anterion vs. Argos (a), Anterion vs. IOLMaster 700 (b), Anterion vs. Pentacam AXL (c), Argos vs. IOLMaster 700 (d), Argos vs. Pentacam AXL (e) and IOLMaster 700 vs. Pentacam AXL (f). The plots show the mean (continuous line), lower and upper limits of agreement (± 1.96 SD [standard deviation], peripheral dotted lines), and the lower and upper confidence intervals (95%). The *P* values are included in each comparison (*significant differences < 0.05).

**Additional file 3: Fig. S3.** Bland–Altman plots of the mean difference versus the average of anterior chamber depth (ACD) used to compare the different devices: Anterion vs. Argos (a), Anterion vs. IOLMaster 700 (b), Anterion vs. Pentacam AXL (c), Argos vs. IOLMaster 700 (d), Argos vs. Pentacam AXL (e) and IOLMaster 700 vs. Pentacam AXL (f). The plots show the mean (continuous line), lower and upper limits of agreement (± 1.96 SD [standard deviation], peripheral dotted lines), and the lower and upper confidence intervals (95%). The *P* values are included in each comparison (*significant differences < 0.05).

**Additional file 4: Fig. S4.** Bland–Altman plots of the mean difference versus the average of lens thickness (LT) used to compare the different devices: Anterion vs. Argos (a), Anterion vs. IOLMaster 700 (b) and Argos vs. IOLMaster 700 (c). The plots show the mean (continuous line), lower and upper limits of agreement (± 1.96 SD [standard deviation], peripheral dotted lines), and the lower and upper confidence intervals (95%). The *P* values are included in each comparison (*significant differences < 0.05).

**Additional file 5: Fig. S5.** Bland–Altman plots of the mean difference versus the average of axial length used to compare the different devices: Anterion vs. Argos (a), Anterion vs. IOLMaster 700 (b), Anterion vs. Pentacam AXL (c), Anterion vs. OcuScan RxP (d), Argos vs. IOLMaster 700 (e), Argos vs. Pentacam AXL (f), Argos vs. OcuScan RxP (g), IOLMaster 700 vs. Pentacam AXL (h), IOLMaster 700 vs. OcuScan RxP (i) and Pentacam AXL vs. OcuScan RxP (j). The plots show the mean (continuous line), lower and upper limits of agreement (± 1.96 SD [standard deviation], peripheral dotted lines), and the lower and upper confidence intervals (95%). The *P* values and number of eyes assessed are included in each comparison (*significant differences < 0.05).

**Additional file 6: Table S1:** Clinical studies that have used optical biometers and reported axial length acquisition success rates in eyes with dense cataracts.

## Data Availability

All data generated or analyzed during this study are included in this published article.
