# Supplementary figures and images for: Axial length acquisition success rates and agreement of four optical biometers and one ultrasound biometer in eyes with dense cataracts
Source: Eye Vis (Lond). 2023 Sep 1;10:35. doi: 10.1186/s40662-023-00352-3 (PMC10472586; doi:10.1186/s40662-023-00352-3)

Mean Difference (mm)

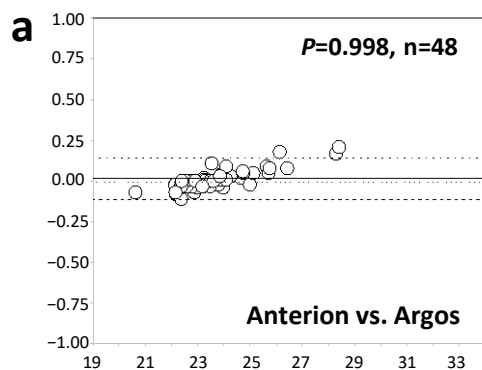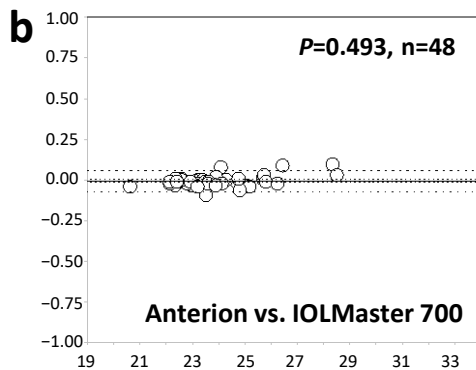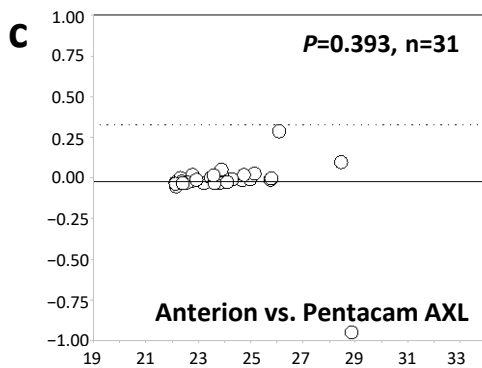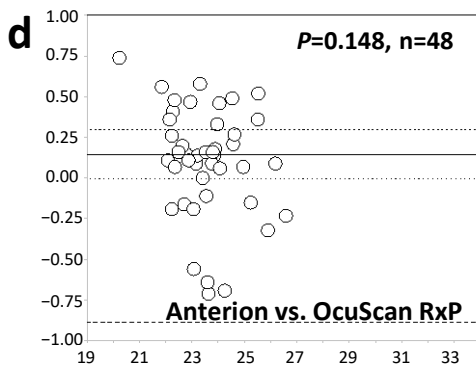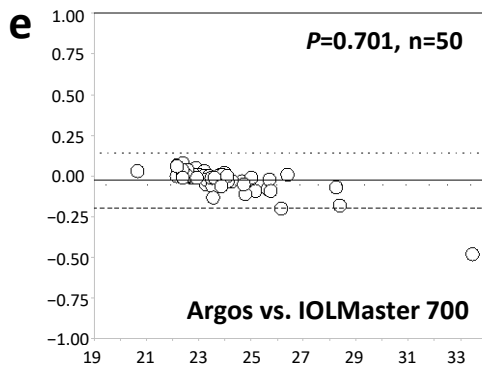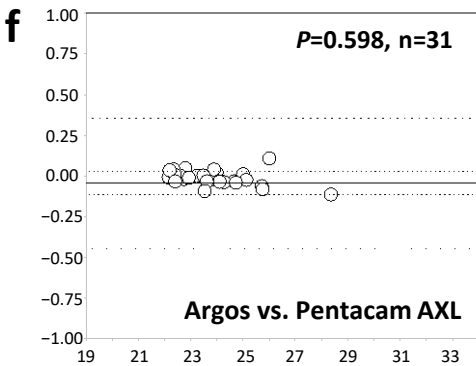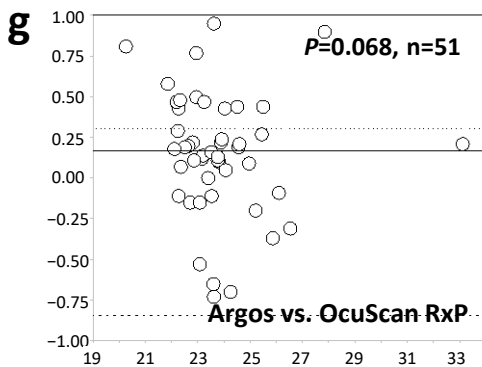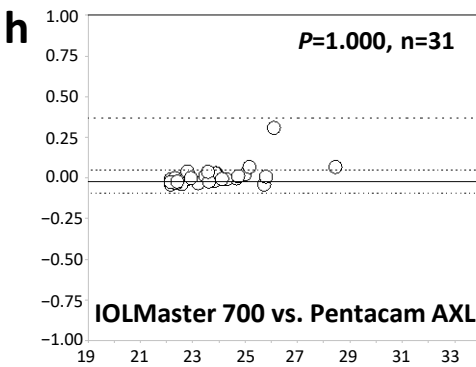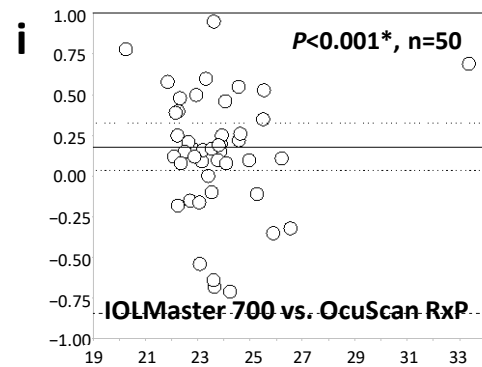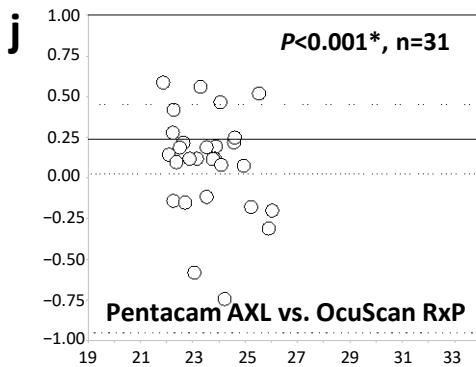

Average Axial Length (mm)

Supplement: Supplementary file 5 — Additional file 5: Fig. S5. Bland–Altman plots of the mean difference versus the average of axial length used to compare the different devices: Anterion vs. Argos (a), Anterion vs. IOLMaster 700 (b), Anterion vs. Pentacam AXL (c), Anterion vs. OcuScan RxP (d), Argos vs. IOLMaster 700 (e), Argos vs. Pentacam AXL (f), Argos vs. OcuScan RxP (g), IOLMaster 700 vs. Pentacam AXL (h), IOLMaster 700 vs. OcuScan RxP (i) and Pentacam AXL vs. OcuScan RxP (j). The plots show the mean (continuous line), lower and upper limits of agreement (± 1.96 SD [standard deviation], peripheral dotted lines), and the lower and upper confidence intervals (95%). The P values and number of eyes assessed are included in each comparison (*significant differences < 0.05). [file 40662_2023_352_MOESM5_ESM.pdf]
