# Supplementary material for: Axial length acquisition success rates and agreement of four optical biometers and one ultrasound biometer in eyes with dense cataracts
Source: Eye Vis (Lond). 2023 Sep 1;10:35. doi: 10.1186/s40662-023-00352-3 (PMC10472586; doi:10.1186/s40662-023-00352-3)
Supplement: Supplementary file 6 — Additional file 6: Table S1: Clinical studies that have used optical biometers and reported axial length acquisition success rates in eyes with dense cataracts. [file 40662_2023_352_MOESM6_ESM.docx]

**Table S1:** Clinical studies that have used optical biometers and reported axial length acquisition success rates in eyes with dense cataracts.

| **Authors** | **Optical biometer** | **Sample (No. of eyes)** | **Age**  **(years)** | **Acquisition rates (%)** | **Cataract type whole sample**  **(eyes)** | **Cataract type unsuccessfully measured**  **(eyes)** |
| --- | --- | --- | --- | --- | --- | --- |
| ***Hirnschall et al.^13^*** | IOLMaster 700 | 23 | 68.4±14.8  (32 to 88) | 91.3 | Nuclear cataract (10), PSC (9) and nuclear + PSC (4) | Nuclear cataract (2) |
| ***Henriquez et al.^14^*** | IOLMaster 700  Galilei G6  Pentacam AXL | 45 | NA | 84.4  42.2  37.7 | *NC: 4.76; NO: 4.96; C: 3.91; P: 3.22 | *NC: 5.45; NO: 5.27; C: 4.64; P: 4.27  *NC: 5.40; NO: 5.30; C: 4.57; P: 4.07  *NC: 5.23; NO: 5.14; C: 4.31; P: 3.71 |
| ***Vasavada et al.^15^*** | Lenstar LS900  OA-2000 | 124† | 58.40±11.14  (27 to 83) | 77.42  98.4 | § Grade 1 (23), grade 2 (30), grade 3 (33), grade 4 (24), grade 5 (14) | White mature, dense PSC with a posterior capsule plaque, or posterior polar cataracts  NA |
| ***Tamaoki et al.^16^*** | Argos  IOLMaster 700  OA-2000 | 99 | NA | 89.9  63.6  80.8 | § Grade ≥4 | Mature or white cataract (10)  Mature or white cataract (29), grade 4 with PSC (3) and without PSC (3)  Mature or white cataract (17), grade 4 without PSC (1) |
| ***Tamaoki et al.^17^*** | Argos  Argos with ERV  IOLMaster 700 | 213 | 71.9±14.8  (NA) | 69.5  93.4  61.5 | § Grade 4 (115), grade 5 (65), white cataracts (33) | NA  NA  White cataracts (25) |
| ***González-Godínez et al.^18^*** | IOLMaster 700  IOLMaster 500 | 70 | 65.21±12.84  (NA) | 78.57  31.43 | *Grades ≥ NC 4, NO 4, C 4, and P 3 | ‡Intumescent cataracts  ‡NO5, NO6, mixed C+P and intumescent cataracts |
| ***Current study*** | Anterion  Argos  IOLMaster 700  Pentacam AXL  OcuScan RxP | 51 | 72.06±8.45  (50 to 89) | 94.12  100  98.04  60.78  100 | *Grade ≥3, DLI ≤5 | *PSC 3 (1), NC 4 (1) and NC 6 (1)  *NC 6 (1)  *NO 3 (5), NO 4 (5), NC 3 (2), NC 4 (1), NC 5 (2), NC 6 (3), PSC 3 (1), PSC 4 (1) |

PSC= posterior subcapsular cataracts; NA= not available; NC= nuclear color; * for LOCS III; NO: nuclear opalescence; C: cortical; P: subcapsular posterior; †: note that this sample includes low-grade cataracts (see cataract type column); §: Emery–Little classification; ERV: enhanced retina visualization mode, ‡: total failure.
